# Cardiovascular Manifestations of COVID-19 Infection

**DOI:** 10.3390/cells9112508

**Published:** 2020-11-19

**Authors:** Ajit Magadum, Raj Kishore

**Affiliations:** 1 Center for Translational Medicine, Temple University, Philadelphia, PA 19140, USA; Ajit.magadum@temple.edu; 2 Department of Pharmacology, Lewis Katz School of Medicine, Temple University, Philadelphia, PA 19140, USA

**Keywords:** COVID-19, SARS-CoV-2, angiotensin converting enzyme-2, cardiovascular disease, myocardial injury, cytokine storm and inflammation

## Abstract

SARS-CoV-2 induced the novel coronavirus disease (COVID-19) outbreak, the most significant medical challenge in the last century. COVID-19 is associated with notable increases in morbidity and death worldwide. Preexisting conditions, like cardiovascular disease (CVD), diabetes, hypertension, and obesity, are correlated with higher severity and a significant increase in the fatality rate of COVID-19. COVID-19 induces multiple cardiovascular complexities, such as cardiac arrest, myocarditis, acute myocardial injury, stress-induced cardiomyopathy, cardiogenic shock, arrhythmias and, subsequently, heart failure (HF). The precise mechanisms of how SARS-CoV-2 may cause myocardial complications are not clearly understood. The proposed mechanisms of myocardial injury based on current knowledge are the direct viral entry of the virus and damage to the myocardium, systemic inflammation, hypoxia, cytokine storm, interferon-mediated immune response, and plaque destabilization. The virus enters the cell through the angiotensin-converting enzyme-2 (ACE2) receptor and plays a central function in the virus’s pathogenesis. A systematic understanding of cardiovascular effects of SARS-CoV2 is needed to develop novel therapeutic tools to target the virus-induced cardiac damage as a potential strategy to minimize permanent damage to the cardiovascular system and reduce the morbidity. In this review, we discuss our current understanding of COVID-19 mediated damage to the cardiovascular system.

## 1. Introduction

COVID-19 (Coronavirus disease of 2019) is caused by infection from severe acute respiratory syndrome coronavirus 2 (SARS-CoV-2) [1,2]. SARS-CoV-2 are single-stranded positive-sense RNA viruses of approximately 30 kb in length, and its virion is 50–200 nm in diameter [1]. Beta coronaviruses infect mammals and COVID-19 is widely considered to have arisen from bats with mutations in the receptor-binding domain (RBD) and the furin protease cleavage site. In humans, the virus infects the upper respiratory (UR) tract and gastrointestinal (GI) tract [2]. Coronaviruses infect human cells via binding of its spike protein to the ACE2 receptors of host cells [2]. SARS-CoV2 invades the cell via receptor-mediated endocytosis by creating the virus’s S protein cleavage by the transmembrane serine protease TMPRSS2 [3,4,5]. SARS-CoV2 replication inside the cells occurs through the RNA-dependent RNA polymerase to encode its structural and functional proteins.

The common symptoms of COVID-19 are fever, cough, shortness of breath or dyspnea, muscle aches, diarrhea, loss of smell and taste, and fatigue in most patients [6]. In some cases, it develops severe acute respiratory distress syndrome (ARDS), CVD, disseminated intravascular coagulation (DIC), and multi-organ failure [3,4,6,7]. Recent literature suggests that COVID-19-infected patients with preexisting CVD have increased severity and a higher fatality rate [5,7,8]. Recent COVID-19 patient studies have shown that persons with CVD, hypertension, coagulation aberrations, and diabetes have severe symptoms and higher mortality rates [3,9,10,11]. In addition to CVD, potential risks also include age, sex, immunosuppressive condition, multi-organ dysfunction, chronic respiratory diseases, renal abnormalities, obesity, and cancer.

It is vital to identify the molecular- and cellular-level interplay between COVID-19 and CVD. This review will compile an existing understanding of the cardiovascular effects of COVID-19. We will also highlight the potential cardiovascular considerations towards developing treatment strategies.

## 2. SARS-CoV-2 Infection

To understand the consequences of SARS-CoV-2 infection on the CV system, it is crucial to study the fundamental biological mechanisms underlying viral entry into the host cells, subsequent immune response, and organ injury. ACE2 is a membrane protein that is highly expressed in the heart, lung, gut, and kidneys and has many physiological functions. It may facilitate damage to the organ by direct virus entry during the course of infection or by a secondary response [12]. A recent single-cell RNA sequencing study showed that more than 7.5% of myocardial cells express ACE2, which could mediate SARS-CoV-2 entry into cardiomyocytes or other ACE2 expressing cells and cause direct cardiotoxicity [13]. SARS-CoV-2 differs from SARS-CoV by more than 380 amino acid substitutions, including six different amino acids in its receptor-binding domain. The host cell proteases, like transmembrane protease serine 2 (TMPRSS2), help in SARS-CoV-2 entry and infection [14]. The binding affinity of SARS-CoV-2 with ACE2 appears stronger than SARS-CoV, which might help for more vital interaction and infectivity. Hence, we see the global pandemic of COVID-19 compared to SARS [15,16]. Moreover, SARS-CoV-2 has evolved to utilize a wide array of host proteases, such as TMPRSS2 for S-protein priming and facilitating enhanced cell entry following receptor binding [17], while the protease inhibitors blocked the entry of SARS-CoV-2 into the cell [18,19]. Therefore SARS-CoV-2 requires co-expression of ACE2 and TMPRSS2 in the same cell type for cell entry and infection [17]. Thus, ACE2 appears to be indispensable for SARS-CoV-2 infection, and its expression in different cells and organs may be predictive of ensuing pathology. For example, ACE2 on type II alveolar epithelial cells allows entry to the virus to develop lung complications, while in pericytes and endothelial cells (EC), viral entry leads to the development of microvascular dysfunction, and disseminated intravascular coagulation (DIC). The virus in cardiomyocyte will likely lead to the cardiac damage and CVD, etc. [20,21]. SARS-CoV-2 enters the cell via receptor-mediated endocytosis, replicates, synthesizes protein, and makes multiple copies of itself to transduce the next cell.

TMPRSS2 and ACE2 help the virus to enter into the cells; however, development of strategies to inhibit these proteins may potentially be utilized for therapeutic purposes aimed at preventing viral entry and consequent severity of the infection [22]. Inhibition of both of these receptors by using chemical inhibitors, antibodies, or siRNA might have an inhibitory effect on viral infection. The use of inhibitors against viral proteins, including its RNA polymerase, is in the clinical study. It was shown that TMPRSS2 inhibition (by protease inhibitor or in TMPRSS2 knock-out mice) prevented viral entry and reduced the viral infection and severity of lung pathology with improved survival after SARS-CoV infection in mouse models [23,24]. Recent discoveries have hinted that the virus might enter the cardiac tissue through ACE2 receptors and damage the heart. Nevertheless, clinical support for the presence of SARS-CoV-2 in the heart of patients with COVID-19 is insufficient, and data on the ubiquitous cardiac viral expression in patients with COVID-19 remains preliminary and evolving [10,25,26].

Recent studies showed the SARS-CoV-2 is transmitted primarily through respiratory droplets by close contact with infected people or surfaces, coughs, and sneezes, like other respiratory coronaviruses, and might be through the fecal-oral route, although evidence for the latter route is scant [6,27]. SARS-CoV-2 median incubation period is around 4–5 days in the host before symptoms arise [28,29,30,31] and most of the patients develop symptoms within 11–12 days [30]. The patients with COVID-19 usually display difficulty in breathing, dry cough, fever, diarrhea, nausea, headache, muscle pain, and dizziness [9,28,32,33,34,35]. SARS-CoV-2 viral load reaches its peak in a shorter time than SARS-CoV in just 5–6 days of symptoms onset [36,37,38]. Severe COVID-19 cases advance to ARDS, cytokine storm, respiratory failure, and CV complications in 8–15 days of symptom onset [4].

## 3. COVID-19 in Heart Disease

**Figure 1:**
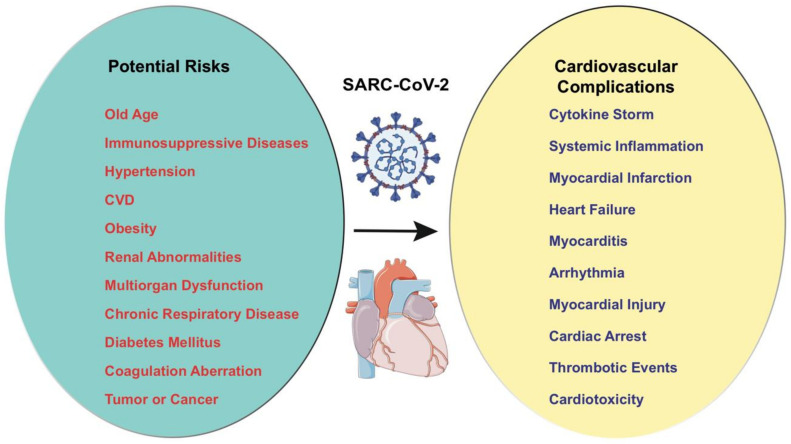
The potential risk factors and the cardiovascular complications in COVID-19. The possible risk factors are old age, immunosuppressive diseases, hypertension, CVD, chronic respiratory disease, obesity, renal abnormalities, diabetes mellitus, coagulation aberrations, and tumor. COVID-19-induced cardiovascular complications can be myocardial infarction, cytokine storm, heart failure, systemic inflammation, myocarditis, arrhythmia, myocardial injury, cardiac arrest, thrombotic events, and cardiotoxicity.

Recent patient data originating from different geographical locations showed that preexisting CVD is associated with a more severe COVID-19 infection [10,25,26]. A recent study by Li et al. showed that, among 1527 COVID-19-infected patients, those with CVD, diabetes, and hypertension were more likely to require ICU admissions [39]. Wu et al., with extensive COVID-19 patient data from China, reported a five-fold increase in the death rate in patients with preexisting CV complications compared with patients without CVD (10.5% vs. 2.3%) [26]. Multiple studies have shown similar findings with an increased risk of mortality in patients with prior CVD. Figure 1 summarizes the relationship between COVID-19 and cardiovascular complications [40,41].

The cardiomyocyte structural protein, troponin (cTnT), level in plasma is a known indicator of cardiac injury. It was recently shown that 54.5% of patients with underlying CVD have higher circulating cTnT levels and were more likely to have cardiac injury and complexities than patients without CVD (13.2%) [40]. Additionally, patients with higher cTnT levels, along with existing CVD, showed higher mortality rates. The higher cTnT levels in patients were more frequently correlated with malignant arrhythmias. In another study, ACE inhibitors or angiotensin receptor blockers were used, but their effect was not significant on the mortality rates. The fatality rates of patients with and without the use of ACE inhibitors/angiotensin receptor blockers were 36.8% and 25.6%. The above observations suggest that patients with pre-existing CVD are prone to more severe complications of COVID-19 with increased mortality but may not be more susceptible to contracting SARS-CoV-2 [41].

**Figure 2:**
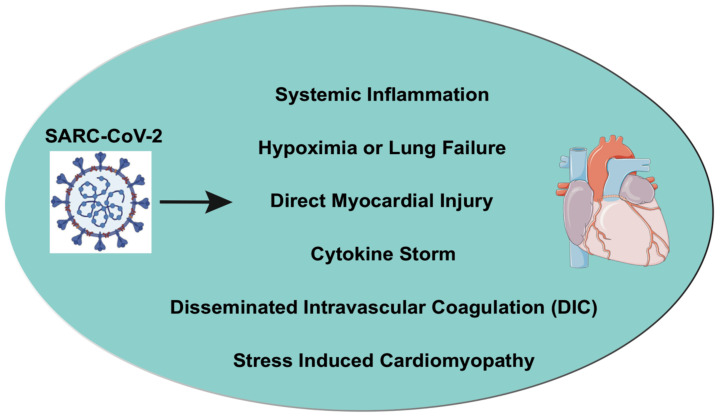
The hypothetical mode of action on heart in COVID-19. COVID-19 can induce cardiovascular complications through different mechanisms, including direct myocardial infection or injury, cytokine storm, hypoxemia or lung failure, systemic inflammation, DIC, and stress-induced cardiomyopathy.

The mechanisms of COVID-19 induced cardiovascular injury have not been fully understood, early clinical data suggesting that CV complications are most likely happening in multiple ways or mechanisms. SARS-CoV-2 can induce direct cardiotoxicity by infecting the cells (cardiomyocytes) and develop myocarditis. The viral particles have been identified in cardiac tissue in some patients [42]. Hyper inflammation post-COVID-19 infection with uncontrolled cytokine release may lead to vascular inflammation, plaque instability, or myocardial inflammation, which further leads to myocardial infarction (MI), cardiomyopathy, and heart failure (HF) [43,44]. COVID-19 infection also induces systemic complications like sepsis and DIC, which may, in turn, also evolve into different cardiac complications. The available epidemiological and histological data combined with symptoms noted with COVID-19 infection suggest that there are certain parallels between COVID-19 and SARS and MERS-induced pathological characteristics in various organs [45,46]. The pathological findings in COVID-19-affected cardiac tissue showed a minor change to interstitial inflammatory infiltration to the hyperactivation of inflammation, cytokine storm, myocyte necrosis, myocarditis, MI, and HF. In the vasculature, microthrombosis and vascular inflammation have also been found. Figure 2 outlines the hypothetical mode of action in COVID-19 on the heart.

## 4. ACE2, CVD and COVID-19

ACE 2 is a type 1 transmembrane protein and has an enzymatic domain that converts angiotensin II into angiotensin [1,2,3,4,5,6,7] on the outer surface of cells [12,47,48]. This enzyme is homologous to ACE but serves a counterbalancing role in the renin-angiotensin-aldosterone system (RAAS). ACE2 is expressed in multiple tissues, including the heart, lungs, and kidneys [12,49]. ACE2 is an essential regulator of cardiac function; it induces a beneficial effect, acts as a vasodilator, anti-fibrotic, anti-oxidative, and anti-hypertrophic [47]. In animal models, ACE2 knockout mice, or its inhibition, develops severe left ventricular dysfunction [50,51]. Beyond its function in cardiovascular physiology and homeostasis, ACE2 is also a functional receptor and entry for both SARS-CoV and SARS-CoV-2. SARS-CoV-2 infection is shown to down-regulate ACE2, which may contribute to myocardial dysfunction [51]. The use of RAAS antagonists may be useful in COVID-19 but, in animal studies, it was shown to increase the ACE2 expression. Thus, increased ACE2 availability might induce further virus infection and susceptibility to infection. However, there are not many studies done in the context of COVID-19.

**Figure 3:**
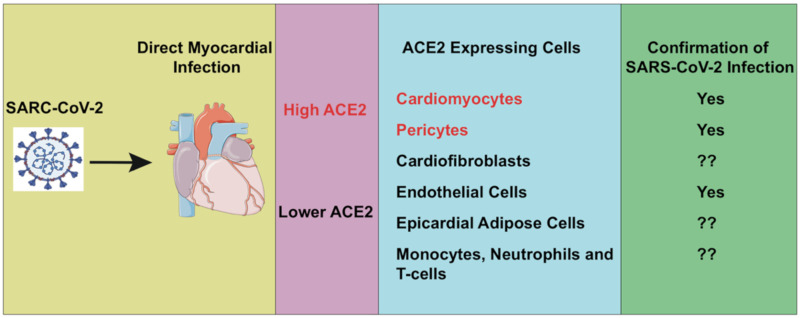
ACE2 expression and SARS-CoV-2 infection of heart cells. The potential SARS-CoV-2 direct infection in heart will be through ACE2-expressing cells, like cardiomyocytes and pericytes (high level expression of ACE2), while cardio-fibroblast, endothelial cells, epicardial adipose cells, and immune cells (low level expression of ACE2) may also be infected.

SARS-CoV-2 enters into cells by interacting with extracellular domains of the transmembrane ACE2 proteins with his viral spike (S)-protein and which is shown to down-regulate the expression of ACE2. It is well known that the down-regulation of ACE2 induces the expression of oligopeptide angiotensin Ang II [18,19,51,52]. Recently a small cohort study of COVID-19 patients showed that the expression of Ang II was significantly increased compared to healthy people; this suggests there is downregulation of ACE2, which results in the systemic RAAS imbalance [53]. Overall this RAAS imbalance induces the development of multi-organ damage from SARS-CoV-2 infections [53]. It is well established, as well as shown by many studies, that various ACE2 polymorphisms are linked to CVD [54]. The ACE2 mRNA expression in human, mice, and rat hearts was significantly increased post-MI [20]. Loss of ACE2 is known to increase neutrophil infiltration in the infarct area, resulting in the cascade of molecular pathway activation to activate the immune response. The expression of pro-inflammatory cytokines, interferon-γ, interleukin (IL)-6, and the chemokine, monocyte chemoattractant protein-1 (MCP-1) were increased significantly, the phosphorylation of ERK1/2 (extracellular signal-regulated kinase ½) and JNK1/2 (c-Jun N-terminal kinase ½) signaling pathways were also elevated. The inhibition or deletion of the ACE2 receptor in mice resulted in larger infarct size, the activation of metalloproteinase like MMP2, MMP9, and disruption of the extracellular matrix culminated in the MI-induced HF [20]. However, the overexpression of the ACE2 receptor or Ang 1–7 improves MI-induced cardiac remodeling in mice [55,56]. Nicin et al. recently showed that ACE2 receptor expression in different types of human heart cells. They used single nuclei RNA sequencing and analyzed the expression of ACE and ACE2 in the single-cell levels in the human heart with aortic stenosis (AS), HF with reduced ejection fraction (HFrEF), and healthy donor hearts. They showed that ACE2 expression was low in fibroblasts, endothelial cells, and leucocytes, with high levels in cardiomyocytes and pericytes [13,57,58]. In comparison, cardiomyocytes from patients with heart disease showed a higher level of ACE2 expression compared to their healthy counterparts. However, ACE2 levels were not significantly changed in endothelial cells and fibroblasts between diseased and healthy subjects. The potential strategy to reduce the COVID-19-induced damage on the heart may include inhibition of SARS-CoV-2 binding to ACE2 by small molecule inhibitors, overexpression of ACE2, recombinant ACE2, ACE2 binding peptide, and ACE2 antibody. Figure 3 summarizes the SARS-CoV-2 infecting cells in the heart.

## 5. Cardiac Injury in COVID-19

There is significantly less known about the molecular and cellular involvement of cardiovascular damage from COVID-19. Multiple studies show that COVID-19 patients with preexisting CV complications were more likely to have a higher level of cardiac troponin (cTnT) elevation in plasma than patients without CVD complications [40]. The cTnT level analysis is vital from a prognostic standpoint where non-survivors had a high level of cTnT, which continued to rise until death, while COVID-19 survivors’ cTnT levels do not change significantly. The monitoring of cTnT levels intermittently in hospitalized COVID-19 patients would help to a great extent. The cTnT levels are known to go up in acute myocardial injury, atherosclerotic plaque disruption, coronary thrombosis, critically ill patients, supply-demand imbalance leading to myocardial injury, infection-based metabolic stress, tachycardia, hypoxia, acidosis, and hypotension [59]. Taken together, these studies suggest that patients with preexisting CV complications are prone to more severe complexities of COVID-19 with high-level death, while they are not significantly susceptible to acquiring SARS-CoV-2 infection compared to healthy people [40,41]. Continued analyses of troponin might be helpful in predicting the risk in patients who are not already critically sick. Table 1 summarizes how the presence of pre-existing morbidities like CVD, hypertension, diabetes, and obesity augment the severity of COVID-19.

It is unclear whether myocardial injury post-COVID-19 reflects a systemic or local and ischemic or inflammatory process. It is still unknown whether an acute injury results from primary infection from the virus or secondary to lung disease or systemic inflammation. As mentioned before, the pathological features, mode of transfection, and mortality of COVID-19 in multiple organs very much parallel those seen in SARS and MERS [45,46]. Recently, the viral particles of SARS-CoV-2 have been identified by RT-PCR in some patients’ heart samples, suggesting the virus’ direct role in cardiac injury. This remains a possibility since cardiomyocytes express a high level of ACE2. In another study, COVID-19 patients with the endo-myocardial biopsy after acute myocardial injury and cardiogenic shock showed mild myocardial inflammation without cardiomyocyte necrosis or apoptosis. These patients showed that the localization of SARS-CoV-2 is not within cardiomyocytes, but it is within macrophages [60]. These data support the idea that SARS-CoV-2 can reside within the heart, but further studies are needed to decipher which cells of the heart get infected directly from the virus.

It was recently shown that human-induced pluripotent stem cell-derived cardiomyocytes (hiPSC-CMs) could be transfected with SARS-CoV-2 in vitro [61]. The ACE2 expression on the surface of cardiomyocytes allowed SARS-CoV-2 to enter, replicate, and produce multiple copies of the virus to infect the other cells. Immunostaining of SARS-CoV-2 unique dsRNA intermediate and the “spike” capsid protein confirmed that the virus entered in hiPSC-CMs and used the host translational machinery to produce new viral components. Next, the author showed the cytotoxic effect of the virus on cell morphology and elevated synthesis of cleaved caspase-3, a marker of apoptosis, suggesting increased cell death through apoptosis. There was a significant decrease in the contractility, or cells ceased beating altogether. Overall, data suggest that SARS-CoV-2 can infect human cardiomyocytes and exert cytotoxic effects.

The drugs (anti-viral, agonist, or antagonists) or therapies used to treat COVID-19 patients for severe multi-organ dysfunction in COVID-19 patients might induce cardiac toxicity. In the case of SARS-CoV-1 infection, it was shown that there is a direct cellular viral infection of the myocardium, and cells reside within the conduction pathways of the heart [51]. With other virus studies, it was demonstrated that in acute myocarditis has direct cellular injury by viral entry into the myocytes, generation of the innate immune response, which leads to myocardial necrosis and damage [62]. As a result of this, there is contractile dysfunction and HF. Virally-driven hyper-inflammation with cytokine storm causes multiple abnormalities in the cardiovascular system, like unstable plaque, vascular inflammation, a hypercoagulable state, myocardial inflammation, and direct myocardial suppression [43,44]. Simultaneously, the systemic ramifications of COVID-19 include sepsis and small blood clots through the body, including vascular inflammation and microthrombosis. Together these multiple events develop CV complications in COVID-19. SARS-CoV infections can cause lung injury, leading to hypoxemia and vasoconstriction, which means decreased oxygen delivery to the heart. This continued reduced supply of oxygen to the heart may lead to myocardial ischemia and subsequent HF or heart disease. DIC is a limited, but critical, condition that causes abnormal blood clotting throughout the body’s blood vessels. DIC develops multi-organ damage through bleeding and thrombosis and is also implicated in heart diseases, including severe cardiac dysfunction, thrombosis of coronary arteries, and myocardium necrosis [63]. There is not much DIC seen in survivors of COVID-19 (0.6%; 1/162), compared to 71.4% (15/21) of non-survivors with COVID-19 [64]. Further studies are required to clarify whether myocardial injury predominately occurs directly due to SARS-CoV-2 cardiomyocyte infection or indirectly due to systemic cytokine release or any other mechanism.

COVID-19 might induce cardiac injury other than the ischemic pathways, including broken heart syndrome or stress cardiomyopathy, acute and fulminant myocarditis [65,66]. A decade ago, it was shown that the SARS-CoV viral RNA was found in autopsied human cardiac samples. Further study showed significant reductions in ACE2 mRNA and protein levels in the cardiac samples, suggesting direct myocardial invasion by SARS-CoV [51]. However, there was a notable myocardial macrophage infiltration of post-mortem heart samples [51]. As a result of ACE-2 down-regulation, there is a significant reduction in angiotensin 1–7, leading to increased TNFα production [67,68]. The down-regulation of ACE2 might amplify to the increased thrombotic risk [69]. Angiotensin 1–7 is known to inhibit the pro-inflammatory cytokines like IL-6, TNFα, IL-12, IL-5, and other inflammatory pathways, like NF-kB (nuclear factor kappa-light-chain-enhancer of activated B cells) and JNK. Many researchers also showed the activation of pro-inflammatory pathways, C-reactive protein, higher inflammatory markers, and elevated cardiomyocyte markers in plasma-like cTnT and cTnI in patients with underlying CVD and high mortality. This supports the notion that the severe inflammatory response in COVID-19 might be a potential mediator of cardiomyocyte damage [40]. Recently, it was shown that significant lung fibrosis occurred by the SARS virus through activation TGF-β-SMAD molecular pathway. This same pathway is a master regulator of fibrosis development in the myocardium [70].

Altogether, with COVID-19 patients’ pathological studies, ACE2 expression in the heart, and significant resemblance of SARS-CoV infection with COVID-19, the possible mechanisms of myocardial injury in COVID-19 could be direct systemic inflammation, exaggerated cytokine response by immune cells, hypoxia, DIC, myocardial fibrosis, and direct damage to the cardiomyocytes. These processes develop into cardiac dysfunction and HF. However, the pathophysiological mechanisms underlying myocardial injury caused by COVID-19 are not well known so far, and most of the data obtained from postmortem tissue or clinical samples are largely observational. Further investigations using controlled cellular or animal models will be required to systematically delineate the exact molecular mechanisms.

## 6. Endothelial Damage in COVID-19

The vascular endothelial cells line the circulatory system and are indispensable for preserving and regulating vascular homeostasis [71]. Endothelial dysfunction is a systemic pathological state that changes the vascular balance towards more pathological conditions, including vasoconstriction, inflammation, tissue edema, organ ischemia, and pro-coagulant state [72]. Vascular smooth muscle cells express both ACE2 receptors and TMPRSS2 protease on cell membranes suggesting there should be a possible direct entry of the virus. Pathological evaluation of different affected organs post COVID-19, including the lungs, has shown microvascular inflammation, together with microvascular thrombi. Second observations, which are early indications of SARS-CoV-2 infection, include distal vasculitis with acrosyndrome and dyshidrosis in terminal digits of patients with COVID-19.

Systemic inflammation, abnormal coagulation status, and multi-organ dysfunction are possible contributing determinants of increased risk for venous thromboembolism (VTE). COVID-19 patients are at an increased risk of VTE [73,74,75]. These patients are reported to exhibit higher D-dimer levels, coagulation pathway abnormalities, and fibrin degradation products associated with increased mortality, which are also features of DIC and pulmonary embolism [12,64,73,76]. DIC is a common phenomenon and has been seen in 71.4% of non-survivors of COVID-19 [64]. The Chinese patient data suggest that a D-dimer elevation is profoundly predictive of unfavorable results in COVID-19. A recent study shows that when patients with severe COVID-19 and having higher D-dimer levels were treated with heparin, they showed reduced mortality, suggesting that anticoagulation therapy might help COVID-19-infected patients, but further studies are needed to confirm this observation [64]. Activated macrophages, either post-injury or in systemic inflammatory diseases, release IL-1β, IL-6, and other pro-inflammatory cytokines, which are known to induce endothelial activation, inflammatory cell infiltration, and vascular inflammation by expression of adhesion molecules. These processes may be enhanced locally if there is a viral infection in adjoining vascular smooth muscle cells. The localized macrophages can release plasminogen activators as pro-coagulant factors. The activation of angiotensin II after the downregulation of ACE2 further accelerates vascular inflammation and enhances a pro-thrombotic state (higher level of IL-6 and d-dimer). The dysfunctional endothelium becomes pro-adhesive and pro-coagulant [77]. The presence of microangiopathy and microthrombi can also predispose the patient to micro-infarcts within multiple organs, such as the heart, liver, or kidney, further exacerbating the state of multi-organ injury and failure.

Recent studies have shown that the presence of viral particles within the endothelial cells and increased endothelial cell death [78] along with the accumulation of inflammatory cells surrounding the viral infected and dead EC. This may explain the systemically-impaired microcirculatory function in patients with COVID-19. These data suggest that SARS-CoV-2 infection induces endotheliitis in several organs as a result of the host inflammatory response and presence of a virus [78]. The inflammation of blood vessels and thrombosis can lead to pulmonary embolism, which worsens hypoxemia, resulting in very low-level oxygen in the blood. The combination of pulmonary embolism with systemic inflammatory or cytokine storm can worsen cardiac injury and HF. The anti-inflammatory or anticoagulation therapies in the initial phase of COVID-19 should be considered in patients with a higher level of IL-6 and D-dimer or DIC, or signs of vasculitis or progressive inflammation.

## 7. Microvascular Coagulopathy and Platelets in COVID-19

Cytokine storm, unrestrained inflammation-mediated endothelial injury, platelets, and renin-angiotensin system dysregulation are involved in COVID-19 induced coagulopathy. Recently, it was reported that a 31% occurrence of thrombotic complications in COVID-19 occurs in patients in the ICU [79]. In another study, it was found that 23% of patients with COVID-19 had an acute pulmonary embolism [80]. Antiphospholipid syndrome (APS) is an autoimmune disorder that can trigger frequent clotting in arteries and veins, leading to increased thrombosis risk. A unique subset of APS is Lupus anticoagulant, anticardiolipin, and anti-β2-glycoprotein characterized as triple-positive antibodies. These antibodies become elevated in viral infections as well as in syphilis and during autoimmune diseases like systemic lupus erythematosus [81]. Recent COVID-19 patient studies showed that the presence of antiphospholipid antibodies in the plasma, and 50% of these patients were positive for Lupus anticoagulant [82]. It is unknown how the antiphospholipid antibodies interact with inflammation or complement activation, resulting in worsened coagulopathy. However, anticoagulants, like heparin, are used as a medication to treat APS, suggesting why heparin may have some beneficial effect on COVID-19 patients. Von Willebrand factor (VWF) is biosynthesized in endothelial cells and megakaryocytes only and is either secreted into the plasma or is stored within intracellular organelles. Recent reports show elevated levels of VWF in COVID-19 patients (more than 565%) [83]. The high VWF concentrations were observed to modulate platelet adhesion and aggregation by binding to platelet receptors [84,85]. This pathological VWF is responsible for thrombotic microangiopathy, which is the symbol of thrombotic thrombocytopenic purpura [86]. Severe COVID-19 patients witnessed similar conditions.

COVID-19 patients showed increased pro-inflammatory cytokine and chemokine levels which, in turn, may disrupt the endothelial function and integrity [3]. This process leads to the upregulation of cell adhesion molecules (ICAM-1, integrin αvβ3, P- and E-selectin), the release of VWF, and induces endothelial production of cytokines and chemokines [87,88]. This results in the pro-inflammatory and procoagulant state that allows the recruitment of platelets and leukocytes [89,90]. In turn, this down-regulates the in situ anticoagulants and an increase in endothelial tissue factors [88]. COVID-19 patients showed increased hypoxia in SARS-CoV-2-infected area. In general, hypoxia activates the pro-thrombotic endothelial state and induces HIFs (hypoxia-inducible transcription factors) in the vascular system which, in turn, down-regulate the natural anticoagulants, Protein S, and TFPI (tissue factor pathway inhibitor) and up-regulates endothelial TF expression, consequently developing a procoagulant endothelial state [91]. ACE2 is known as the primary receptor used by SARS-CoV2 for cellular entry in humans. However, recently, several other cell receptors were found that enable SARS-CoV-2 entry into the cells. These include CD209L, CD209 [92], Neuropilin receptors (NRPs) [93,94] and CD147/Basigin [95]. In general, viruses exercise many mechanisms for attachment and cell entry. Cell adhesion molecules are among the most prevalent receptors viruses use for infection [96].

Recently, Giuseppe Lippi et al. pooled data from 1779 COVID-19 patients, including 22.4% with severe cases with decreased platelet counts, suggesting this may be associated with an increased risk of death [97]. Platelets are quite abundant in the vasculature, mediating hemostasis and regulating immune functions [98,99]. Stimulated platelets release plenty of chemokines from their granules, like CXCL1 (chemokine (C-X-C motif) ligand), PF-4 (platelet factor-4), CXCL5, CXCL7 (chemokine (C-C motif) ligand), and CCL3, CCL5, and CCL7 [100]. These released chemokines are known to boost leukocyte recruitment to vascular injury/inflammation sites. Patients with COVID-19 exhibit high levels of neutrophil extracellular traps (NETs) in the serum released by platelets; NETs are networks of extracellular fibers made of DNA from neutrophils. NETosis is an exacerbation of tissue injury, inflammation, and intravascular thrombosis. The sera of COVID-19 patients produced more NETosis in purified neutrophils obtained from patients on ventilation compared to patients with mild symptoms, suggesting that levels of NETs correlate with enhanced disease severity [101]. The activated platelets derived NETs in COVID-19 patients induce FXII to generate thrombin, and this might induce the proactive thrombotic state observed in COVID-19. Further study is required to define the role of platelets in COVID-19. COVID-19 patients also showed higher complement pathway activation. This complement activation leads to enhanced production of endothelial-production of cytokines, such as IL-1, IL-8, RANTES, IL-6, and MCP-1, and up-regulates crucial endothelial adhesion molecules, such as P-selectin and VWF, which further helps to develop thrombin formation in COVID-19 patients [102]. Hence, the elevated levels of endothelial cytokines and adhesion molecules, higher D-dimer, and NETs observed in COVID-19 patients show the thrombin generation’s dysregulation, which is a feature of the prothrombotic nature in COVID-19.

## 8. Oxidative Stress and Apoptosis in COVID-19

At low to moderate concentration, ROS is known to be indispensable for the regulation of normal physiological functions involved in the development and homeostasis, like cell differentiation, cell cycle and proliferation, migration, and cell death. Oxidative stress has been shown to perform a pivotal role in the pathophysiology of cardiac remodeling and HF. Oxidative stress is nothing but excess production of reactive oxygen species (ROS) relative to the antioxidant defense mechanism of a system. The increased ROS derived from mitochondria and enzymes like NAD(P)H oxidase, xanthine oxidase, and uncoupled nitric oxide synthase lead to mitochondrial DNA damage as well as functional decline, which in turn induce further ROS generation. ROS directly modifies proteins involved in excitation-contraction coupling to impair contractile function and develop contractile dysfunction. Increased ROS is known to stimulate cardiac fibroblast proliferation and activate the matrix metalloproteinases, leading to extracellular matrix remodeling and fibrosis. ROS also induce the apoptotic pathways post-heart injury and in pathophysiologic condition. ROS is known to activate different hypertrophy signaling pathways [103].

Previously, it was shown that cardiotropic virus enters into the cell, especially CMs, and then induces an innate immune response that, in turn, induces local or global myocardial necrosis [62]. Within a few days of this, direct injury leads to clinical symptoms, like contractile dysfunction and HF. SARS-CoV-1 stays in the heart for around five days but, in some studies, it was shown that the virus might persist for months [104]. We expect similar pathophysiology with the SARS-CoV-2 virus in the heart. Recently, with multiple studies, it has been speculated that there is an ischemic effect on the heart other than direct infection of the virus. The ischemic effect occurs in two ways, one by hypoxic (ischemia) situation by lung pathology post virus infection, and second is the direct toxicity by the virus at the macro- or microvascular level. Long-time viral stimulation induces intense immunological reactions, cytokine storm, and immune-cell infiltration. The ROS can be synthesized in excess by infected cells, including the CM’s EC and even immune cells, and can produce H_2_O_2_, (·O2 −), (·OH), etc. [105,106]. It is well known that ROS is essential in regulating immunological responses in different conditions, including the clearing of viruses. However, excessive ROS may oxidize the cellular macromolecules, like proteins and lipids, resulting in the prompt destruction of not only virus-infected cells but also healthy cells that may lead to multiple organ failure. As endothelial cells express ACE2, virus infection-induced vascular damage by endothelial shedding and dysfunction may enhance ROS production and local inflammation, and production of coagulant factors and thrombosis. Overall, these processes may contribute to the risk of myocardial damage, and these processes will be similar to the increase in myocardial infarctions observed after viral infections like influenza [107].

Although studies focused on the direct role of oxidative stress and its biomarkers in COVID-19 patient samples are not yet reported, there are ample examples of the indirect role of oxidative stress linked to SARS-CoV-2-induced multiple manifestations that have been reported [3,33,42,53,64,73,108]. This review highlights systemic oxidative stress as a potential consequence of multiple biological activities following COVID-19 infection that may exacerbate cardiac damage. Numerous antioxidants (ROS scavengers) have been tested in clinical trials to reduce the oxidative burden of cardiovascular diseases resulting in mixed outcomes. While ROS scavengers are effective at reducing cellular ROS levels, they are, in general, ineffective in cardiovascular pathology. It is essential to study whether an intrinsically defective anti-oxidant system, such as G6PD deficiency or other causes, is activated in response to COVID-19 infection [109,110]. Moreover, the use of anti-oxidant therapy may help in inhibiting COVID-19 pathology [111,112,113].

## 9. Inflammation and Cytokine Storm Induced by COVID-19

In the early infection phase of COVID-19, the virus infiltrates the lung parenchyma and begins to proliferate extremely rapidly, yet many patients are asymptomatic, with mild natural indications and signs showing an initial response by innate immunity, specifically monocytes and macrophages. While the immune system is mounting a response, it is not sufficient to attenuate the viral replication potential. Simultaneous tissue injury and the inflammatory processes result in vasodilation, endothelial permeability, and leukocyte recruitment, which results in further damage to the lung, hypoxic condition, and cardiovascular stress. In a subset of patients, the host inflammatory response continues to amplify and results in systemic inflammation [114,115]. These systemic complications have the potential to further injure multiple distant organs, including the heart and kidney. There have been patients with myocardial complications without evidence of direct viral infiltration, implicating the heart as one such target of systemic inflammation [42]. Fulminant myocarditis (FM) is characterized by sudden and severe diffuse cardiac inflammation, often leading to death. There are reports of FM seen in patients with viral infection, including SARS-CoV-2, and myocardial inflammation, which may play a role in cardiac injury, however, the mechanism of SARS-CoV2-mediated FM is unclear [5,65,66,116].

Evaluating lymphocyte-mediated myocardial injury in SARS-CoV-2 infection remains a topic of interest. There is limited understanding about the presence of SARS-CoV or SARS-CoV-2 epitopes in the myocardium in the scientific community, while many of the epitopes against spike (S) and nucleocapsid (N) proteins of SARS-CoV have been observed in the peripheral blood mononuclear cells [117,118]. Multiple recent studies have shown that COVID-19 induces a strong cytokine storm and multi-organ damage; however, there is no robust evidence of direct lymphocytic infiltration in the myocardium. However, a current study of 150 patients infected with SARS-CoV-2 showed a 100% increase in the level of ferritin and IL-6 in non-survivors as opposed to the survivors [119,120]. Simultaneously, serum levels of IL-6, IL-10, IL-2R, and TNF-α were raised in patients with severe disease [33]. This systemic release of cytokines characterized by increased pro-inflammatory cytokines likely contributes to the dysregulation of T cells and the ensuing systemic inflammation that leads to cardiac injury and multi-organ damage. Inhibitors of interleukins, specifically IL-6 inhibitor tocilizumab administration, has shown improvement related to decreased cardiovascular complications. Therefore, tocilizumab might be potentially helpful in the case of COVID-19 infection, although this needs to be validated in randomized clinical trials.

It is well established that the functions of both innate and adaptive immunity decline with aging. A study in mice showed that in comparison to the SARS-CoV-MA15-infected young C57BL/6 mice, infection of 12-months aged mice was linked with a severe decrease in the number of virus-specific CD8+ T cells in the lungs [121]. Lesser severity in infection and effect of SARS-CoV-2 in children may be linked to a better equipped immune system. Furthermore, gender appears to impart a protective effect on COVID-19 infection and severity. Older males showed a higher incidence of SARS-CoV-2 infection compared to females [9]. Despite existing literature on gender differences in immune or inflammatory responses, the exact molecular underpinnings of this disparity are not well understood. Hence, additional studies are needed to confirm any conclusions on age- and gender-dependent risk for cytokine storm and subsequent cardiac injury in COVID-19 infection.

Cytokine storm or cytokine release syndrome is characterized by significantly enhanced production of pro-inflammatory cytokines, such as IL-1, IL-6, interferon-gamma, and TNF-alpha. The body develops a cytokine storm in response to multiple pathologies, including injuries and viral infections such as infection with the SARS virus [122,123,124]. Similarly, SARS-CoV-2 can elicit the intense release of multiple cytokines and chemokines by the host immune system [3,124]. There is substantial data that patients with severe COVID-19 can develop cytokine storm and increased plasma levels of interleukins multiply pro-inflammatory cytokines [3]. The cytokine storm noted in some COVID-19 patients could have profound CV consequences and may cause the onset of tachycardia, hypotension, and left ventricular dysfunction. It is claimed that pro-inflammatory cytokines dampen myocardial function instantly by activation of different signaling pathways, specifically the neural sphingomyelinase pathway, and acutely via nitric oxide-mediated blunting of beta-adrenergic signaling [125]. Cytokine storm has also been correlated with direct cardiotoxicity and may lead to the development of conduction abnormalities, atrial fibrillation, and sustained cardiac injury characterized by elevated levels of BNP and cardiac markers in plasma [126].

The immune organs were also significantly affected by COVID-19. The spleen and lymph nodes showed a very significant reduction in the number of lymphocytes (CD4+ and CD8+). Similarly, there was a reduction in the number of neutrophils, as well as cell death. The FACS analyses of T-cells in COVID-19 active cases have also shown the hyperactivation of T lymphocytes, suggesting the uncontrolled overactivation of T cells may account for, in part, the severe immune injury [108]. Previous multiple studies show that SARS-CoV infects T lymphocytes, and the virus particles were seen in T lymphocytes, macrophages, and dendritic cells derived from monocytes [127,128,129]. The viral infection in these immune cells, especially in monocytes and macrophages, can induce an anomalous cytokine expression and contribute to cytokine storm, while virus entry into lymphocytes could reduce lymphocytes and develop lymphopenia in patients. The extent to which SARS-CoV-2 infects these cells remains crudely explained. The precise knowledge of the molecular and cellular mechanisms of immune dysfunction in COVID-19 is critical for the development of suitable anti-immune or immune-modulatory therapeutics.

## 10. Limitations of Animal Models

The perfect pre-clinical animal model, which simulates human disease in the context of the route and spread of infection, the symptoms and molecular mechanism, the severity of the disease, and relative levels of mortality, is required to investigate molecular mechanisms of the disease. The similarity should be seen in viral receptor expression, viral replication, pathogenesis, and disease or complication severity similar to that in humans. We need the right model for the right question and study. Recent studies have shown that ferrets, non-human primates, hamsters, and cats are highly susceptible to SARS-CoV-2; dogs have low susceptibility, while pigs, chickens, and ducks are not susceptible to the virus.

Rodents, including mice, provide disease models that have several beneficial characteristics. The mouse model is easy to handle and breed, is relatively low cost, and is amenable to easy and accurate manipulation at the genetic level. Mice are useful in evaluating the virus entry into the host, the pathogenesis of viruses, and molecular and cellular changes in the host. Mice can also be efficiently used for the development of preliminary testing of vaccines and anti-viral drugs. However, mice shrug off infection with SARS-CoV-2 quickly because the mouse ACE2 to which SARS-CoV-2 first attaches has 11 of 29 amino acids of domain different from the human version. Few mouse models were developed in the past to study SARS-CoV, which were based on hACE2-transgenic mice (human ACE2), adapting human coronavirus and immunodeficient mice [130]. Recently, a novel mouse model for COVID-19 was generated using the delivery of human ACE2 with a replication-deficient adenovirus (Ad5-hACE2). These mice, when exposed to the SARS-CoV2, showed high-titer virus replication in the lungs, developed a pneumonia-like condition, and demonstrated severe pulmonary pathology, weight loss, and Type I interferon response. These mice showed increased inflammation, and innate and adaptive immune response. The T-cell response was strong (CD4 and CD8 transcripts were upregulated), and there was increased B-cell and macrophage infiltration. Several cytokines and chemokines, including TNF, were upregulated, consistent with observations in COVID-19 patients. This mouse model may help in the rapid assessment of a vaccine candidate, anti-viral therapies, and of human convalescent plasma therapies for COVID-19 treatment [131]. However, no notable infection or histopathological injuries were observed in the other organs, including the heart, in these mice. The data suggests the hACE2-transgenic mouse may not fully recapitulate the severe and fatal cases of COVID-19 in humans. In the last decade, tissue organoids were developed as a suitable and alternative system to explore virus replication pathogenesis and therapeutic studies [132]. Recently, multiple studies showed different tissue organoids (intestinal organoids, human blood vessel, human kidney, prostate, retina, brain, and lung organoids) could be used as in vitro organ systems to mimic the SARS-CoV-2 viral infection, pathogenesis, and ACE2 expression, and were studied for therapeutic effects (drugs, proteins, or nucleic acid therapeutics) [133,134,135].

As this mouse model did not show the SARS-CoV-2-induced cardiovascular complication like what we see in human patients or samples, it is difficult to use them for cardiovascular-specific effects. However, the development of mouse models that mimic cardiovascular complications would be significantly valuable. The expression of hACE2 using different promoters, enhancers, cell specificity, and expression levels with or without endogenous mouse ACE2 expression will help to develop a better COVID-19 pathogenesis model. The in vitro cell culture and tissue models will also be helpful in studying SARS-CoV-2 infection, cytotoxicity, molecular mechanisms, and drug or therapy effects on cells. As multiple studies have shown, the iPSC-derived cardiomyocytes could be transfected by SARS-CoV-2 and develop cytotoxic effects and death [61,136,137,138,139,140]. The use of cardiac organoids will be instructive to mimic COVID-19 infection of the heart but, as of now, there is no study available. The use of hACE2 mice with pre-existing cardiovascular risks, like hypertension, cardiovascular diseases, obesity, diabetes, and coagulation aberrations, will be helpful to develop severe COVID-19 model and develop therapy against these cardiovascular complications.

## 11. Conclusions

In January 2020, the World Health Organization (WHO) declared COVID-19 an international emergency. Multiple studies have shown that COVID-19 is associated with several cardiovascular complications, including myocardial injury and myocarditis, acute MI, HF, arrhythmias, stress-induced cardiomyopathy, VTE, acute coronary syndrome, and DIC. There are concerns regarding how medications utilized to treat COVID-19 patients may lead to cardiovascular complications, and their use should be carefully considered before treating COVID-19 patient. Several efforts have already been made to understand the diagnosis and prognosis of these cardiovascular complications, but the data is limited. The exact mechanisms of COVID-19-induced cardiovascular complications are unclear but likely involves a combination of processes, which includes direct viral infection, hypoxemia, hyperinflation, immune response, cytokine storm, DIC, and stress-induced cardiomyopathy.

There remains a multitude of questions that are not yet answered due to limited studies relying on cardiovascular clinical datasets on COVID-19 exposure: (1) What are the genetic and non-genetic factors that influence the susceptibility, severity, and clinical outcomes in COVID-19? (2) What are the molecular and cellular mechanisms of cardiovascular complications induced post-COVID-19? (3) What are the therapies or treatments that may protect against COVID-19-induced cardiovascular complications? There are more than 100 vaccine development initiatives based on modified mRNA, adenovirus-associated virus (DNA), and inactivated virus running worldwide, and dozens of them are in clinical phases 1 and 2, while there are 11 vaccine programs in clinical phase 3 [141,142,143,144,145,146]. While preliminary human clinical trials showed humoral and T-cell response after a vaccine injection, however, there are significant questions regarding the effects of these vaccines and their successful development with strong immunity in humans against SARS-CoV-2 infection. It is difficult to predict how long these vaccines will protect people from SARS-CoV-2 infection. The immune protection might be for months or years rather than the rest of one’s life, and the virus might develop resistance against it. Ongoing research and development of animal models to recapitulate human disease, particularly with the emphasis on cardiovascular effects of COVID-19, will hopefully shed new light on these and other questions.

## Figures and Tables

**Table 1:** Baseline characteristics of patients who tested positive for COVID-19 and compared with CVD, hypertension, diabetes, and obesity occurrence. Data are reported as absolute numbers and/or in percentages (%).

Sr. No.	Study	Sample Numbers	Death	CVD	Hypertension	Diabetes Mellitus	Obesity
1	Garg et al.	178	n/a	27.80%	49.70%	28.30%	n/a
2	Shi et al.	416	n/a	14.70%	30.50%	14%	n/a
3	Grasselli et al.	1043	n/a	21.40%	48.80%	21.40%	n/a
4	Bello-Chavolla OY et al.	51,633	5332 (10.3)	1381 (2.7%)	11,151 (21.6)	9460 (18.3%)	10708 (20.7%)
5	Argenziano et al.	1000	n/a	233 (23.3)	601 (60.1%)	372 (37.2%)	352 (35.2%)
6	Hajifathalian et al.	770	n/a	162 (21.0%)	432 (56.1%)	238 (30.9%)	277 (35.9%)
7	Price-Haywood et al.	3481	n/a	267 (7.6%)	1074 (30.8%)	566 (16.2%)	1727 (49.6%)
8	Huang et al.	41	n/a	15%	15%	20%	n/a
9	Wang et al.	138	n/a	15%	31%	10%	n/a
10	Zhou et al.	191	54	8%	30%	19%	n/a
11	Liu et al.	78	n/a	3%	40%	25%	n/a
12	Guan et al.	1099	n/a	3%	15%	7%	n/a

CVD, cardiovascular disease; n/a not available.

## References

[1] Graham R.L., Donaldson E.F., Baric R.S. (2013). A decade after SARS: Strategies for controlling emerging coronaviruses. Nat. Rev. Microbiol..

[2] Su S., Wong G., Shi W., Liu J., Lai A.C.K., Zhou J., Liu W., Bi Y., Gao G.F. (2016). Epidemiology, Genetic Recombination, and Pathogenesis of Coronaviruses. Trends Microbiol..

[3] Huang C., Wang Y., Li X., Ren L., Zhao J., Hu Y., Zhang L., Fan G., Xu J., Gu X. (2020). Clinical features of patients infected with 2019 novel coronavirus in Wuhan, China. Lancet.

[4] Wang D., Hu B., Hu C., Zhu F., Liu X., Zhang J., Wang B., Xiang H., Cheng Z., Xiong Y. (2020). Clinical Characteristics of 138 Hospitalized Patients with 2019 Novel Coronavirus-Infected Pneumonia in Wuhan, China. JAMA.

[5] Zeng J.H., Liu Y.X., Yuan J., Wang F.X., Wu W.B., Li J.X., Wang L.F., Gao H., Wang Y., Dong C.F. (2020). First case of COVID-19 complicated with fulminant myocarditis: A case report and insights. Infection.

[6] Zhou F., Yu T., Du R., Fan G., Liu Y., Liu Z., Xiang J., Wang Y., Song B., Gu X. (2020). Clinical course and risk factors for mortality of adult inpatients with COVID-19 in Wuhan, China: A retrospective cohort study. Lancet.

[7] Xu Z., Shi L., Wang Y., Zhang J., Huang L., Zhang C., Liu S., Zhao P., Liu H., Zhu L. (2020). Pathological findings of COVID-19 associated with acute respiratory distress syndrome. Lancet Respir. Med..

[8] Fang L., Karakiulakis G., Roth M. (2020). Are patients with hypertension and diabetes mellitus at increased risk for COVID-19 infection?. Lancet Respir. Med..

[9] Chen N., Zhou M., Dong X., Qu J., Gong F., Han Y., Qiu Y., Wang J., Liu Y., Wei Y. (2020). Epidemiological and clinical characteristics of 99 cases of 2019 novel coronavirus pneumonia in Wuhan, China: A descriptive study. Lancet.

[10] Murthy S., Gomersall C.D., Fowler R.A. (2020). Care for Critically Ill Patients with COVID-19. JAMA.

[11] Weiss P., Murdoch D.R. (2020). Clinical course and mortality risk of severe COVID-19. Lancet.

[12] Donoghue M., Hsieh F., Baronas E., Godbout K., Gosselin M., Stagliano N., Donovan M., Woolf B., Robison K., Jeyaseelan R. (2000). A novel angiotensin-converting enzyme-related carboxypeptidase (ACE2) converts angiotensin I to angiotensin 1-9. Circ. Res..

[13] Nicin L., Abplanalp W.T., Mellentin H., Kattih B., Tombor L., John D., Schmitto J.D., Heineke J., Emrich F., Arsalan M. (2020). Cell type-specific expression of the putative SARS-CoV-2 receptor ACE2 in human hearts. Eur. Heart J..

[14] Wu A., Peng Y., Huang B., Ding X., Wang X., Niu P., Meng J., Zhu Z., Zhang Z., Wang J. (2020). Genome Composition and Divergence of the Novel Coronavirus (2019-nCoV) Originating in China. Cell Host Microbe.

[15] Yan R., Zhang Y., Li Y., Xia L., Guo Y., Zhou Q. (2020). Structural basis for the recognition of SARS-CoV-2 by full-length human ACE2. Science.

[16] Shang J., Ye G., Shi K., Wan Y., Luo C., Aihara H., Geng Q., Auerbach A., Li F. (2020). Structural basis of receptor recognition by SARS-CoV-2. Nature.

[17] Millet J.K., Whittaker G.R. (2015). Host cell proteases: Critical determinants of coronavirus tropism and pathogenesis. Virus Res..

[18] Li W., Moore M.J., Vasilieva N., Sui J., Wong S.K., Berne M.A., Somasundaran M., Sullivan J.L., Luzuriaga K., Greenough T.C. (2003). Angiotensin-converting enzyme 2 is a functional receptor for the SARS coronavirus. Nature.

[19] Walls A.C., Park Y.J., Tortorici M.A., Wall A., McGuire A.T., Veesler D. (2020). Structure, Function, and Antigenicity of the SARS-CoV-2 Spike Glycoprotein. Cell.

[20] Burrell L.M., Risvanis J., Kubota E., Dean R.G., MacDonald P.S., Lu S., Tikellis C., Grant S.L., Lew R.A., Smith A.I. (2005). Myocardial infarction increases ACE2 expression in rat and humans. Eur. Heart J..

[21] Kassiri Z., Zhong J., Guo D., Basu R., Wang X., Liu P.P., Scholey J.W., Penninger J.M., Oudit G.Y. (2009). Loss of angiotensin-converting enzyme 2 accelerates maladaptive left ventricular remodeling in response to myocardial infarction. Circ. Heart Fail..

[22] Bertram S., Heurich A., Lavender H., Gierer S., Danisch S., Perin P., Lucas J.M., Nelson P.S., Pohlmann S., Soilleux E.J. (2012). Influenza and SARS-coronavirus activating proteases TMPRSS2 and HAT are expressed at multiple sites in human respiratory and gastrointestinal tracts. PLoS ONE.

[23] Iwata-Yoshikawa N., Okamura T., Shimizu Y., Hasegawa H., Takeda M., Nagata N. (2019). TMPRSS2 Contributes to Virus Spread and Immunopathology in the Airways of Murine Models after Coronavirus Infection. J. Virol..

[24] Zhou Y., Vedantham P., Lu K., Agudelo J., Carrion R., Nunneley J.W., Barnard D., Pohlmann S., McKerrow J.H., Renslo A.R. (2015). Protease inhibitors targeting coronavirus and filovirus entry. Antivir. Res..

[25] Driggin E., Madhavan M.V., Bikdeli B., Chuich T., Laracy J., Biondi-Zoccai G., Brown T.S., Der Nigoghossian C., Zidar D.A., Haythe J. (2020). Cardiovascular Considerations for Patients, Health Care Workers, and Health Systems during the COVID-19 Pandemic. J. Am. Coll. Cardiol..

[26] Wu Z., McGoogan J.M. (2020). Characteristics of and Important Lessons from the Coronavirus Disease 2019 (COVID-19) Outbreak in China: Summary of a Report of 72314 Cases from the Chinese Center for Disease Control and Prevention. JAMA.

[27] Kucharski A.J., Russell T.W., Diamond C., Liu Y., Edmunds J., Funk S., Eggo R.M. (2020). Centre for Mathematical Modelling of Infectious Diseases COVID-19 Working Group. Early dynamics of transmission and control of COVID-19: A mathematical modelling study. Lancet Infect. Dis.

[28] Guan W.J., Ni Z.Y., Hu Y., Liang W.H., Ou C.Q., He J.X., Liu L., Shan H., Lei C.L., Hui D.S.C. (2020). Clinical Characteristics of Coronavirus Disease 2019 in China. N. Engl. J. Med..

[29] Pung R., Chiew C.J., Young B.E., Chin S., Chen M.I., Clapham H.E., Cook A.R., Maurer-Stroh S., Toh M., Poh C. (2020). Investigation of three clusters of COVID-19 in Singapore: Implications for surveillance and response measures. Lancet.

[30] Lauer S.A., Grantz K.H., Bi Q., Jones F.K., Zheng Q., Meredith H.R., Azman A.S., Reich N.G., Lessler J. (2020). The Incubation Period of Coronavirus Disease 2019 (COVID-19) from Publicly Reported Confirmed Cases: Estimation and Application. Ann. Intern. Med..

[31] Li Q., Guan X., Wu P., Wang X., Zhou L., Tong Y., Ren R., Leung K.S.M., Lau E.H.Y., Wong J.Y. (2020). Early Transmission Dynamics in Wuhan, China, of Novel Coronavirus-Infected Pneumonia. N. Engl. J. Med..

[32] Chan J.F., Yuan S., Kok K.H., To K.K., Chu H., Yang J., Xing F., Liu J., Yip C.C., Poon R.W. (2020). A familial cluster of pneumonia associated with the 2019 novel coronavirus indicating person-to-person transmission: A study of a family cluster. Lancet.

[33] Chen G., Wu D., Guo W., Cao Y., Huang D., Wang H., Wang T., Zhang X., Chen H., Yu H. (2020). Clinical and immunological features of severe and moderate coronavirus disease 2019. J. Clin. Investig..

[34] Liu Y., Yang Y., Zhang C., Huang F., Wang F., Yuan J., Wang Z., Li J., Li J., Feng C. (2020). Clinical and biochemical indexes from 2019-nCoV infected patients linked to viral loads and lung injury. Sci. China Life Sci..

[35] Phan L.T., Nguyen T.V., Luong Q.C., Nguyen T.V., Nguyen H.T., Le H.Q., Nguyen T.T., Cao T.M., Pham Q.D. (2020). Importation and Human-to-Human Transmission of a Novel Coronavirus in Vietnam. N. Engl. J. Med..

[36] Pan Y., Zhang D., Yang P., Poon L.L.M., Wang Q. (2020). Viral load of SARS-CoV-2 in clinical samples. Lancet Infect. Dis..

[37] Kim J.Y., Ko J.H., Kim Y., Kim Y.J., Kim J.M., Chung Y.S., Kim H.M., Han M.G., Kim S.Y., Chin B.S. (2020). Viral Load Kinetics of SARS-CoV-2 Infection in First Two Patients in Korea. J. Korean Med. Sci..

[38] Zou L., Ruan F., Huang M., Liang L., Huang H., Hong Z., Yu J., Kang M., Song Y., Xia J. (2020). SARS-CoV-2 Viral Load in Upper Respiratory Specimens of Infected Patients. N. Engl. J. Med..

[39] Li B., Yang J., Zhao F., Zhi L., Wang X., Liu L., Bi Z., Zhao Y. (2020). Prevalence and impact of cardiovascular metabolic diseases on COVID-19 in China. Clin. Res. Cardiol. Off. J. Ger. Card. Soc..

[40] Guo T., Fan Y., Chen M., Wu X., Zhang L., He T., Wang H., Wan J., Wang X., Lu Z. (2020). Cardiovascular Implications of Fatal Outcomes of Patients with Coronavirus Disease 2019 (COVID-19). JAMA Cardiol..

[41] Shi S., Qin M., Shen B., Cai Y., Liu T., Yang F., Gong W., Liu X., Liang J., Zhao Q. (2020). Association of Cardiac Injury with Mortality in Hospitalized Patients with COVID-19 in Wuhan, China. JAMA Cardiol..

[42] Yao X.H., Li T.Y., He Z.C., Ping Y.F., Liu H.W., Yu S.C., Mou H.M., Wang L.H., Zhang H.R., Fu W.J. (2020). A pathological report of three COVID-19 cases by minimal invasive autopsies. Zhonghua Bing Li Xue Za Zhi.

[43] Prabhu S.D. (2004). Cytokine-induced modulation of cardiac function. Circ. Res..

[44] Levi M., van der Poll T., Buller H.R. (2004). Bidirectional relation between inflammation and coagulation. Circulation.

[45] Ding Y., Wang H., Shen H., Li Z., Geng J., Han H., Cai J., Li X., Kang W., Weng D. (2003). The clinical pathology of severe acute respiratory syndrome (SARS): A report from China. J. Pathol..

[46] Alsaad K.O., Hajeer A.H., Al Balwi M., Al Moaiqel M., Al Oudah N., Al Ajlan A., AlJohani S., Alsolamy S., Gmati G.E., Balkhy H. (2018). Histopathology of Middle East respiratory syndrome coronovirus (MERS-CoV) infection-clinicopathological and ultrastructural study. Histopathology.

[47] Patel V.B., Zhong J.C., Grant M.B., Oudit G.Y. (2016). Role of the ACE2/Angiotensin 1–7 Axis of the Renin-Angiotensin System in Heart Failure. Circ. Res..

[48] Tipnis S.R., Hooper N.M., Hyde R., Karran E., Christie G., Turner A.J. (2000). A human homolog of angiotensin-converting enzyme. Cloning and functional expression as a captopril-insensitive carboxypeptidase. J. Biol. Chem..

[49] Zou X., Chen K., Zou J., Han P., Hao J., Han Z. (2020). Single-cell RNA-seq data analysis on the receptor ACE2 expression reveals the potential risk of different human organs vulnerable to 2019-nCoV infection. Front. Med..

[50] Crackower M.A., Sarao R., Oudit G.Y., Yagil C., Kozieradzki I., Scanga S.E., Oliveira-dos-Santos A.J., da Costa J., Zhang L., Pei Y. (2002). Angiotensin-converting enzyme 2 is an essential regulator of heart function. Nature.

[51] Oudit G.Y., Kassiri Z., Jiang C., Liu P.P., Poutanen S.M., Penninger J.M., Butany J. (2009). SARS-coronavirus modulation of myocardial ACE2 expression and inflammation in patients with SARS. Eur. J. Clin. Investig..

[52] Zhou P., Yang X.L., Wang X.G., Hu B., Zhang L., Zhang W., Si H.R., Zhu Y., Li B., Huang C.L. (2020). A pneumonia outbreak associated with a new coronavirus of probable bat origin. Nature.

[53] Wang K., Gheblawi M., Oudit G.Y. (2020). Angiotensin Converting Enzyme 2: A Double-Edged Sword. Circulation.

[54] Patel S.K., Velkoska E., Freeman M., Wai B., Lancefield T.F., Burrell L.M. (2014). From gene to protein-experimental and clinical studies of ACE2 in blood pressure control and arterial hypertension. Front. Physiol..

[55] Qi Y.F., Zhang J., Wang L., Shenoy V., Krause E., Oh S.P., Pepine C.J., Katovich M.J., Raizada M.K. (2016). Angiotensin-converting enzyme 2 inhibits high-mobility group box 1 and attenuates cardiac dysfunction post-myocardial ischemia. J. Mol. Med..

[56] Wang Y., Qian C., Roks A.J., Westermann D., Schumacher S.M., Escher F., Schoemaker R.G., Reudelhuber T.L., van Gilst W.H., Schultheiss H.P. (2010). Circulating rather than cardiac angiotensin-(1-7) stimulates cardioprotection after myocardial infarction. Circ. Heart Fail..

[57] Patel V.B., Zhong J.C., Fan D., Basu R., Morton J.S., Parajuli N., McMurtry M.S., Davidge S.T., Kassiri Z., Oudit G.Y. (2014). Angiotensin-converting enzyme 2 is a critical determinant of angiotensin II-induced loss of vascular smooth muscle cells and adverse vascular remodeling. Hypertension.

[58] Patel V.B., Mori J., McLean B.A., Basu R., Das S.K., Ramprasath T., Parajuli N., Penninger J.M., Grant M.B., Lopaschuk G.D. (2016). ACE2 Deficiency Worsens Epicardial Adipose Tissue Inflammation and Cardiac Dysfunction in Response to Diet-Induced Obesity. Diabetes.

[59] Babuin L., Vasile V.C., Rio Perez J.A., Alegria J.R., Chai H.S., Afessa B., Jaffe A.S. (2008). Elevated cardiac troponin is an independent risk factor for short- and long-term mortality in medical intensive care unit patients. Crit. Care Med..

[60] Hoffmann M., Kleine-Weber H., Schroeder S., Kruger N., Herrler T., Erichsen S., Schiergens T.S., Herrler G., Wu N.H., Nitsche A. (2020). SARS-CoV-2 Cell Entry Depends on ACE2 and TMPRSS2 and Is Blocked by a Clinically Proven Protease Inhibitor. Cell.

[61] Sharma A., Garcia G., Arumugaswami V., Svendsen C.N. (2020). Human iPSC-Derived Cardiomyocytes Are Susceptible to SARS-CoV-2 Infection. bioRxiv Prepr. Serv. Biol..

[62] Cooper L.T. (2009). Myocarditis. N. Engl. J. Med..

[63] Sugiura M., Hiraoka K., Ohkawa S., Ueda K., Matsuda T. (1977). A clinicopathological study on cardiac lesions in 64 cases of disseminated intravascular coagulation. Jpn. Heart J..

[64] Tang N., Li D., Wang X., Sun Z. (2020). Abnormal coagulation parameters are associated with poor prognosis in patients with novel coronavirus pneumonia. J. Thromb. Haemost..

[65] Chen C., Zhou Y., Wang D.W. (2020). SARS-CoV-2: A potential novel etiology of fulminant myocarditis. Herz.

[66] Chen L., Li X., Chen M., Feng Y., Xiong C. (2020). The ACE2 expression in human heart indicates new potential mechanism of heart injury among patients infected with SARS-CoV-2. Cardiovasc. Res..

[67] Zhong J., Basu R., Guo D., Chow F.L., Byrns S., Schuster M., Loibner H., Wang X.H., Penninger J.M., Kassiri Z. (2010). Angiotensin-converting enzyme 2 suppresses pathological hypertrophy, myocardial fibrosis, and cardiac dysfunction. Circulation.

[68] Zhong J., Guo D., Chen C.B., Wang W., Schuster M., Loibner H., Penninger J.M., Scholey J.W., Kassiri Z., Oudit G.Y. (2011). Prevention of angiotensin II-mediated renal oxidative stress, inflammation, and fibrosis by angiotensin-converting enzyme 2. Hypertension.

[69] Lupi L., Adamo M., Inciardi R.M., Metra M. (2020). ACE2 down-regulation may contribute to the increased thrombotic risk in COVID-19. Eur. Heart J..

[70] Zhao X., Nicholls J.M., Chen Y.G. (2008). Severe acute respiratory syndrome-associated coronavirus nucleocapsid protein interacts with Smad3 and modulates transforming growth factor-beta signaling. J. Biol. Chem..

[71] Flammer A.J., Anderson T., Celermajer D.S., Creager M.A., Deanfield J., Ganz P., Hamburg N.M., Luscher T.F., Shechter M., Taddei S. (2012). The assessment of endothelial function: From research into clinical practice. Circulation.

[72] Bonetti P.O., Lerman L.O., Lerman A. (2003). Endothelial dysfunction: A marker of atherosclerotic risk. Arter. Thromb. Vasc. Biol..

[73] Danzi G.B., Loffi M., Galeazzi G., Gherbesi E. (2020). Acute pulmonary embolism and COVID-19 pneumonia: A random association?. Eur. Heart J..

[74] Evans P.C., Ed Rainger G., Mason J.C., Guzik T.J., Osto E., Stamataki Z., Neil D., Hoefer I.E., Fragiadaki M., Waltenberger J. (2020). Endothelial Dysfunction in COVID-19: A Position Paper of the ESC Working Group for Atherosclerosis and Vascular Biology, and the ESC Council of Basic Cardiovascular Science. Cardiovasc. Res..

[75] Fauvel C., Weizman O., Trimaille A., Mika D., Pommier T., Pace N., Douair A., Barbin E., Fraix A., Bouchot O. (2020). Pulmonary embolism in COVID-19 patients: A French multicentre cohort study. Eur. Heart J..

[76] Ramlall V., Thangaraj P.M., Meydan C., Foox J., Butler D., Kim J., May B., De Freitas J.K., Glicksberg B.S., Mason C.E. (2020). Immune complement and coagulation dysfunction in adverse outcomes of SARS-CoV-2 infection. Nat. Med..

[77] Boisrame-Helms J., Kremer H., Schini-Kerth V., Meziani F. (2013). Endothelial dysfunction in sepsis. Curr. Vasc. Pharmacol..

[78] Varga Z., Flammer A.J., Steiger P., Haberecker M., Andermatt R., Zinkernagel A.S., Mehra M.R., Schuepbach R.A., Ruschitzka F., Moch H. (2020). Endothelial cell infection and endotheliitis in COVID-19. Lancet.

[79] Klok F.A., Kruip M., van der Meer N.J.M., Arbous M.S., Gommers D., Kant K.M., Kaptein F.H.J., van Paassen J., Stals M.A.M., Huisman M.V. (2020). Incidence of thrombotic complications in critically ill ICU patients with COVID-19. Thromb. Res..

[80] Grillet F., Behr J., Calame P., Aubry S., Delabrousse E. (2020). Acute Pulmonary Embolism Associated with COVID-19 Pneumonia Detected by Pulmonary CT Angiography. Radiology.

[81] Uthman I.W., Gharavi A.E. (2002). Viral infections and antiphospholipid antibodies. Semin. Arthritis Rheum..

[82] Zhang Y., Xiao M., Zhang S., Xia P., Cao W., Jiang W., Chen H., Ding X., Zhao H., Zhang H. (2020). Coagulopathy and Antiphospholipid Antibodies in Patients with Covid-19. N. Engl. J. Med..

[83] O’Sullivan J.M., Gonagle D.M., Ward S.E., Preston R.J.S., O’Donnell J.S. (2020). Endothelial cells orchestrate COVID-19 coagulopathy. Lancet Haematol..

[84] Goshua G., Pine A.B., Meizlish M.L., Chang C.H., Zhang H., Bahel P., Baluha A., Bar N., Bona R.D., Burns A.J. (2020). Endotheliopathy in COVID-19-associated coagulopathy: Evidence from a single-centre, cross-sectional study. Lancet Haematol..

[85] Llitjos J.F., Leclerc M., Chochois C., Monsallier J.M., Ramakers M., Auvray M., Merouani K. (2020). High incidence of venous thromboembolic events in anticoagulated severe COVID-19 patients. J. Thromb. Haemost..

[86] Klok F.A., Kruip M., van der Meer N.J.M., Arbous M.S., Gommers D., Kant K.M., Kaptein F.H.J., van Paassen J., Stals M.A.M., Huisman M.V. (2020). Confirmation of the high cumulative incidence of thrombotic complications in critically ill ICU patients with COVID-19: An updated analysis. Thromb. Res..

[87] Kayal S., Jais J.P., Aguini N., Chaudiere J., Labrousse J. (1998). Elevated circulating E-selectin, intercellular adhesion molecule 1, and von Willebrand factor in patients with severe infection. Am. J. Respir. Crit. Care Med..

[88] Aird W.C. (2003). The role of the endothelium in severe sepsis and multiple organ dysfunction syndrome. Blood.

[89] Massberg S., Enders G., Leiderer R., Eisenmenger S., Vestweber D., Krombach F., Messmer K. (1998). Platelet-endothelial cell interactions during ischemia/reperfusion: The role of P-selectin. Blood.

[90] Springer T.A. (1994). Traffic signals for lymphocyte recirculation and leukocyte emigration: The multistep paradigm. Cell.

[91] Gupta N., Zhao Y.Y., Evans C.E. (2019). The stimulation of thrombosis by hypoxia. Thromb. Res..

[92] Amraie R., Napoleon M.A., Yin W., Berrigan J., Suder E., Zhao G., Olejnik J., Gummuluru S., Muhlberger E., Chitalia V. (2020). CD209L/L-SIGN and CD209/DC-SIGN act as receptors for SARS-CoV-2 and are differentially expressed in lung and kidney epithelial and endothelial cells. bioRxiv.

[93] Daly J.L., Simonetti B., Antón-Plágaro C., Williamson M.K., Shoemark D.K., Simón-Gracia L., Klein K., Bauer M., Hollandi R., Greber U.F. (2020). Neuropilin-1 is a host factor for SARS-CoV-2 infection. bioRxiv.

[94] Cantuti-Castelvetri L., Ojha R., Pedro L.D., Djannatian M., Franz J., Kuivanen S., Kallio K., Kaya T., Anastasina M., Smura T. (2020). Neuropilin-1 facilitates SARS-CoV-2 cell entry and provides a possible pathway into the central nervous system. bioRxiv.

[95] Wang K., Chen W., Zhou Y.-S., Lian J.-Q., Zhang Z., Du P., Gong L., Zhang Y., Cui H.-Y., Geng J.-J. (2020). SARS-CoV-2 invades host cells via a novel route: CD147-spike protein. bioRxiv.

[96] Kerr J.R. (1999). Cell adhesion molecules in the pathogenesis of and host defence against microbial infection. Mol. Pathol..

[97] Lippi G., Plebani M., Henry B.M. (2020). Thrombocytopenia is associated with severe coronavirus disease 2019 (COVID-19) infections: A meta-analysis. Clin. Chim. Acta.

[98] Semple J.W., Italiano J.E., Freedman J. (2011). Platelets and the immune continuum. Nat. Rev. Immunol..

[99] McFadyen J.D., Kaplan Z.S. (2015). Platelets are not just for clots. Transfus. Med. Rev..

[100] Engelmann B., Massberg S. (2013). Thrombosis as an intravascular effector of innate immunity. Nat. Rev. Immunol..

[101] Zuo Y., Yalavarthi S., Shi H., Gockman K., Zuo M., Madison J.A., Blair C., Weber A., Barnes B.J., Egeblad M. (2020). Neutrophil extracellular traps in COVID-19. JCI Insight.

[102] Foley J.H., Conway E.M. (2016). Cross Talk Pathways between Coagulation and Inflammation. Circ. Res..

[103] Hori M., Nishida K. (2008). Oxidative stress and left ventricular remodelling after myocardial infarction. Cardiovasc. Res..

[104] Kuhl U., Pauschinger M., Noutsias M., Seeberg B., Bock T., Lassner D., Poller W., Kandolf R., Schultheiss H.P. (2005). High prevalence of viral genomes and multiple viral infections in the myocardium of adults with “idiopathic” left ventricular dysfunction. Circulation.

[105] Loffredo L., Martino F., Zicari A.M., Carnevale R., Battaglia S., Martino E., Cammisotto V., Peruzzi M., De Castro G., Duse M. (2019). Enhanced NOX-2 derived oxidative stress in offspring of patients with early myocardial infarction. Int. J. Cardiol..

[106] Imai Y., Kuba K., Neely G.G., Yaghubian-Malhami R., Perkmann T., van Loo G., Ermolaeva M., Veldhuizen R., Leung Y.H., Wang H. (2008). Identification of oxidative stress and Toll-like receptor 4 signaling as a key pathway of acute lung injury. Cell.

[107] Waxman D.A., Kanzaria H.K., Schriger D.L. (2018). Acute Myocardial Infarction after Laboratory-Confirmed Influenza Infection. N. Engl. J. Med..

[108] Shi Y., Wang Y., Shao C., Huang J., Gan J., Huang X., Bucci E., Piacentini M., Ippolito G., Melino G. (2020). COVID-19 infection: The perspectives on immune responses. Cell Death Differ..

[109] Jain S.K., Parsanathan R., Levine S.N., Bocchini J.A., Holick M.F., Vanchiere J.A. (2020). The potential link between inherited G6PD deficiency, oxidative stress, and vitamin D deficiency and the racial inequities in mortality associated with COVID-19. Free Radic. Biol. Med..

[110] Polonikov A. (2020). Endogenous Deficiency of Glutathione as the Most Likely Cause of Serious Manifestations and Death in COVID-19 Patients. ACS Infect. Dis..

[111] Fauci A.S., Lane H.C., Redfield R.R. (2020). Covid-19-Navigating the Uncharted. N. Engl. J. Med..

[112] Kornfeld O.S., Hwang S., Disatnik M.H., Chen C.H., Qvit N., Mochly-Rosen D. (2015). Mitochondrial reactive oxygen species at the heart of the matter: New therapeutic approaches for cardiovascular diseases. Circ. Res..

[113] Kassi E.N., Papavassiliou K.A., Papavassiliou A.G. (2020). Defective Anti-oxidant System: An Aggravating Factor for COVID-19 Patients Outcome?. Arch. Med. Res..

[114] Channappanavar R., Perlman S. (2017). Pathogenic human coronavirus infections: Causes and consequences of cytokine storm and immunopathology. Semin. Immunopathol..

[115] Peiris J.S., Chu C.M., Cheng V.C., Chan K.S., Hung I.F., Poon L.L., Law K.I., Tang B.S., Hon T.Y., Chan C.S. (2003). Clinical progression and viral load in a community outbreak of coronavirus-associated SARS pneumonia: A prospective study. Lancet.

[116] Hu H., Ma F., Wei X., Fang Y. (2020). Coronavirus fulminant myocarditis saved with glucocorticoid and human immunoglobulin. Eur. Heart J..

[117] Xu X., Gao X. (2004). Immunological responses against SARS-coronavirus infection in humans. Cell. Mol. Immunol..

[118] Wang Y.D., Sin W.Y., Xu G.B., Yang H.H., Wong T.Y., Pang X.W., He X.Y., Zhang H.G., Ng J.N., Cheng C.S. (2004). T-cell epitopes in severe acute respiratory syndrome (SARS) coronavirus spike protein elicit a specific T-cell immune response in patients who recover from SARS. J. Virol..

[119] Ruan Q., Yang K., Wang W., Jiang L., Song J. (2020). Clinical predictors of mortality due to COVID-19 based on an analysis of data of 150 patients from Wuhan, China. Intensiv. Care Med..

[120] Mehta P., McAuley D.F., Brown M., Sanchez E., Tattersall R.S., Manson J.J. (2020). Hlh across speciality collaboration, U.K. COVID-19: Consider cytokine storm syndromes and immunosuppression. Lancet.

[121] Xu X., Han M., Li T., Sun W., Wang D., Fu B., Zhou Y., Zheng X., Yang Y., Li X. (2020). Effective treatment of severe COVID-19 patients with tocilizumab. Proc. Natl. Acad. Sci. USA.

[122] Wang Z., Zhang A., Wan Y., Liu X., Qiu C., Xi X., Ren Y., Wang J., Dong Y., Bao M. (2014). Early hypercytokinemia is associated with interferon-induced transmembrane protein-3 dysfunction and predictive of fatal H7N9 infection. Proc. Natl. Acad. Sci. USA.

[123] Wong C.K., Lam C.W., Wu A.K., Ip W.K., Lee N.L., Chan I.H., Lit L.C., Hui D.S., Chan M.H., Chung S.S. (2004). Plasma inflammatory cytokines and chemokines in severe acute respiratory syndrome. Clin. Exp. Immunol..

[124] Kim E.S., Choe P.G., Park W.B., Oh H.S., Kim E.J., Nam E.Y., Na S.H., Kim M., Song K.H., Bang J.H. (2016). Clinical Progression and Cytokine Profiles of Middle East Respiratory Syndrome Coronavirus Infection. J. Korean Med. Sci..

[125] Mann D.L. (2015). Innate immunity and the failing heart: The cytokine hypothesis revisited. Circ. Res..

[126] Jamal F.A., Khaled S.K. (2020). The Cardiovascular Complications of Chimeric Antigen Receptor T Cell Therapy. Curr. Hematol. Malig. Rep..

[127] Gu J., Gong E., Zhang B., Zheng J., Gao Z., Zhong Y., Zou W., Zhan J., Wang S., Xie Z. (2005). Multiple organ infection and the pathogenesis of SARS. J. Exp. Med..

[128] Cheung C.Y., Poon L.L., Ng I.H., Luk W., Sia S.F., Wu M.H., Chan K.H., Yuen K.Y., Gordon S., Guan Y. (2005). Cytokine responses in severe acute respiratory syndrome coronavirus-infected macrophages in vitro: Possible relevance to pathogenesis. J. Virol..

[129] Law H.K., Cheung C.Y., Ng H.Y., Sia S.F., Chan Y.O., Luk W., Nicholls J.M., Peiris J.S., Lau Y.L. (2005). Chemokine up-regulation in SARS-coronavirus-infected, monocyte-derived human dendritic cells. Blood.

[130] Bao L., Deng W., Huang B., Gao H., Liu J., Ren L., Wei Q., Yu P., Xu Y., Qi F. (2020). The pathogenicity of SARS-CoV-2 in hACE2 transgenic mice. Nature.

[131] Sun J., Zhuang Z., Zheng J., Li K., Wong R.L., Liu D., Huang J., He J., Zhu A., Zhao J. (2020). Generation of a Broadly Useful Model for COVID-19 Pathogenesis, Vaccination, and Treatment. Cell.

[132] Ettayebi K., Crawford S.E., Murakami K., Broughman J.R., Karandikar U., Tenge V.R., Neill F.H., Blutt S.E., Zeng X.L., Qu L. (2016). Replication of human noroviruses in stem cell-derived human enteroids. Science.

[133] Lamers M.M., Beumer J., van der Vaart J., Knoops K., Puschhof J., Breugem T.I., Ravelli R.B.G., van Schayck J.P., Mykytyn A.Z., Duimel H.Q. (2020). SARS-CoV-2 productively infects human gut enterocytes. Science.

[134] Mahalingam R., Dharmalingam P., Santhanam A., Kotla S., Davuluri G., Karmouty-Quintana H., Ashrith G., Thandavarayan R.A. (2020). Single-cell RNA sequencing analysis of SARS-CoV-2 entry receptors in human organoids. J. Cell. Physiol..

[135] Monteil V., Kwon H., Prado P., Hagelkruys A., Wimmer R.A., Stahl M., Leopoldi A., Garreta E., Del Pozo C.H., Prosper F. (2020). Inhibition of SARS-CoV-2 Infections in Engineered Human Tissues Using Clinical-Grade Soluble Human ACE2. Cell.

[136] Li Y., Renner D.M., Comar C.E., Whelan J.N., Reyes H.M., Cardenas-Diaz F.L., Truitt R., Tan L.H., Dong B., Alysandratos K.D. (2020). SARS-CoV-2 induces double-stranded RNA-mediated innate immune responses in respiratory epithelial derived cells and cardiomyocytes. bioRxiv Prepr. Serv. Biol..

[137] Perez-Bermejo J.A., Kang S., Rockwood S.J., Simoneau C.R., Joy D.A., Ramadoss G.N., Silva A.C., Flanigan W.R., Li H., Nakamura K. (2020). SARS-CoV-2 infection of human iPSC-derived cardiac cells predicts novel cytopathic features in hearts of COVID-19 patients. bioRxiv Prepr. Serv. Biol..

[138] Lu D., Chatterjee S., Xiao K., Riedel I., Wang Y., Foo R., Bar C., Thum T. (2020). MicroRNAs targeting the SARS-CoV-2 entry receptor ACE2 in cardiomyocytes. J. Mol. Cell. Cardiol..

[139] Kwon Y., Nukala S.B., Srivastava S., Miyamoto H., Ismail N.I., Rehman J., Ong S.B., Lee W.H., Ong S.G. (2020). Detection of Viral RNA Fragments in Human iPSC-Cardiomyocytes following Treatment with Extracellular Vesicles from SARS-CoV-2 Coding-Sequence-Overexpressing Lung Epithelial Cells. bioRxiv Prepr. Serv. Biol..

[140] Yang L., Han Y., Nilsson-Payant B.E., Gupta V., Wang P., Duan X., Tang X., Zhu J., Zhao Z., Jaffre F. (2020). A Human Pluripotent Stem Cell-based Platform to Study SARS-CoV-2 Tropism and Model Virus Infection in Human Cells and Organoids. Cell Stem. Cell.

[141] Yang J., Wang W., Chen Z., Lu S., Yang F., Bi Z., Bao L., Mo F., Li X., Huang Y. (2020). A vaccine targeting the RBD of the S protein of SARS-CoV-2 induces protective immunity. Nature.

[142] Zost S.J., Gilchuk P., Case J.B., Binshtein E., Chen R.E., Nkolola J.P., Schafer A., Reidy J.X., Trivette A., Nargi R.S. (2020). Potently neutralizing and protective human antibodies against SARS-CoV-2. Nature.

[143] Gao Q., Bao L., Mao H., Wang L., Xu K., Yang M., Li Y., Zhu L., Wang N., Lv Z. (2020). Development of an inactivated vaccine candidate for SARS-CoV-2. Science.

[144] Jackson L.A., Anderson E.J., Rouphael N.G., Roberts P.C., Makhene M., Coler R.N., McCullough M.P., Chappell J.D., Denison M.R., Stevens L.J. (2020). An mRNA Vaccine against SARS-CoV-2—Preliminary Report. N. Engl. J. Med..

[145] Zhu F.C., Guan X.H., Li Y.H., Huang J.Y., Jiang T., Hou L.H., Li J.X., Yang B.F., Wang L., Wang W.J. (2020). Immunogenicity and safety of a recombinant adenovirus type-5-vectored COVID-19 vaccine in healthy adults aged 18 years or older: A randomised, double-blind, placebo-controlled, phase 2 trial. Lancet.

[146] Braun J., Loyal L., Frentsch M., Wendisch D., Georg P., Kurth F., Hippenstiel S., Dingeldey M., Kruse B., Fauchere F. (2020). SARS-CoV-2-reactive T cells in healthy donors and patients with COVID-19. Nature.

